# [^68^Ga]Ga-Pentixafor for PET Imaging of Vascular Expression of CXCR-4 as a Marker of Arterial Inflammation in HIV-Infected Patients: A Comparison with ^18^F[FDG] PET Imaging

**DOI:** 10.3390/biom10121629

**Published:** 2020-12-03

**Authors:** Ismaheel O. Lawal, Gbenga O. Popoola, Johncy Mahapane, Jens Kaufmann, Cindy Davis, Honest Ndlovu, Letjie C. Maserumule, Kgomotso M. G. Mokoala, Hakim Bouterfa, Hans-Jürgen Wester, Jan Rijn Zeevaart, Mike M. Sathekge

**Affiliations:** 1 Department of Nuclear Medicine, University of Pretoria, Pretoria 0001, South Africa; ismaheellawal@gmail.com (I.O.L.); ndlovuhonest@gmail.com (H.N.); letjie.maserumule@gmail.com (L.C.M.); kmgmokoala@gmail.com (K.M.G.M.); 2 Nuclear Medicine Research Infrastructure (NuMeRI), Steve Biko Academic Hospital, Pretoria 0001, South Africa; janrijn.zeevaart@necsa.co.za; 3 Department of Epidemiology and Community Health, University of Ilorin, Ilorin 240102, Nigeria; g.popoola45@gmail.com; 4 Department of Nuclear Medicine, Steve Biko Academic Hospital, Pretoria 0001, South Africa; jkmahapane@gmail.com (J.M.); sbahtherapy@gmail.com (C.D.); 5 PentixaPharm GmbH, 97082 Wuerzburg, Germany; jens.kaufmann@1717lsv.com (J.K.); hakim.bouterfa@1717lsv.com (H.B.); 6 Pharmazeutische Radiochemie, Technische Universität München, 85748 Garching, Germany; h.j.wester@tum.de; 7 Radiochemistry, South African Nuclear Energy Corporation SOC (Necsa), Pelindaba 0204, South Africa

**Keywords:** HIV infection, [^68^Ga]Ga-pentixafor PET/CT, [^18^F]FDG PET/CT, arterial inflammation, atherosclerotic cardiovascular diseases

## Abstract

People living with human immunodeficiency virus (PLHIV) have excess risk of atherosclerotic cardiovascular disease (ASCVD). Arterial inflammation is the hallmark of atherogenesis and its complications. In this study we aimed to perform a head-to-head comparison of fluorine-18 fluorodeoxyglucose positron emission tomography/computed tomography ([^18^F]FDG PET/CT) and Gallium-68 pentixafor positron emission tomography/computed tomography [^68^Ga]Ga-pentixafor PET/CT for quantification of arterial inflammation in PLHIV. We prospectively recruited human immunodeficiency virus (HIV)-infected patients to undergo [^18^F]FDG PET/CT and [^68^Ga]Ga-pentixafor PET/CT within two weeks of each other. We quantified the levels of arterial tracer uptake on both scans using maximum standardized uptake value (SUVmax) and target–background ratio. We used Bland and Altman plots to measure the level of agreement between tracer quantification parameters obtained on both scans. A total of 12 patients were included with a mean age of 44.67 ± 7.62 years. The mean duration of HIV infection and mean CD+ T-cell count of the study population were 71.08 ± 37 months and 522.17 ± 260.33 cells/µL, respectively. We found a high level of agreement in the quantification variables obtained using [^18^F]FDG PET and [^68^Ga]Ga-pentixafor PET. There is a good level of agreement in the arterial tracer quantification variables obtained using [^18^F]FDG PET/CT and [^68^Ga]Ga-pentixafor PET/CT in PLHIV. This suggests that [^68^Ga]Ga-pentixafor may be applied in the place of [^18^F]FDG PET/CT for the quantification of arterial inflammation.

## 1. Introduction

The risk of atherosclerotic cardiovascular disease (ASCVD) is twice as high in people living with human immunodeficiency virus (PLHIV) compared with people without human immunodeficiency virus (HIV) infection [1]. Factors responsible for the excess risk of ASCVD among PLHIV include the impact of the virus itself, the atherogenic effect of antiretroviral therapy (ART) used in HIV treatment, and preponderance of traditional cardiovascular risk factors among PLHIV [2]. Inflammation is a hallmark feature of atherogenesis and its progression [3]. Atherogenesis evolve over decades and endothelial dysfunction characterized by invasion of vascular endothelium by inflammatory cells is present long before atheroma develops. The critical role inflammation plays in atheroma formation and its progression informs the new interest in developing therapeutic agents that inhibit inflammation in attempt to stem the tide of the increasing incidence of ASCVD [4]. Radionuclide imaging techniques are viable options for non-invasive quantification of arterial inflammation to assess patient risk for ASCVD and to directly monitor the impact of anti-inflammatory therapy.

Flourine-18 fluorodeoxyglucose positron emission tomography ([^18^F]FDG PET) imaging is the most used radionuclide technique for arterial inflammation quantification. In an early study, Subramanian et al., using arterial [^18^F]FDG uptake as a surrogate marker of inflammation, reported higher arterial inflammation in patients with HIV compared with their age-, gender-, and Framingham risk score-matched controls [5]. In the study, arterial inflammation in HIV infected patient was comparable to that in older HIV-uninfected patients with established ASCVD. Our group has also recently reported a higher level of arterial inflammation among young PLHIV without risk for ASCVD except HIV infection compared with age- and gender-matched HIV-uninfected controls [6]. Despite the huge experience with the use of [^18^F]FDG PET for arterial inflammation imaging, there are several limitations associated with it including the need for extensive patient preparation. More importantly, physiologic [^18^F]FDG uptake in the myocardium and structures in the neck makes accurate quantification of its arterial uptake difficult. There is, therefore, a need to evaluate other radionuclide tracers without these shortcomings for their use in arterial inflammation imaging.

Gallium-68 pentixafor ([^68^Ga]Ga-Pentixafor) is a novel tracer that targets chemokine receptor-4 (CXCR-4) expressed on inflammatory and cancer cells. In a proof of concept, Hyafil et al. demonstrated specific localization of [^68^Ga]Ga-Pentixafor to macrophage-rich atheromatous lesions [7]. The specificity of radiolabeled pentixafor for CXCR4 was demonstrated in a blocking experiment with AMD3100 [7]. Weiberg et al. have shown correlations between the level of [^68^Ga]Ga-Pentixafor in arterial lesions and ASCVD risk factors [8]. No study has evaluated the utility of [^68^Ga]Ga-Pentixafor for quantification of arterial inflammation in PLHIV or performed a prospective head-to-head comparison between it and [^18^F]FDG PET imaging. The aim of our study was to report our preliminary experience on a head-to-head comparison of [^68^Ga]Ga-Pentixafor and [^18^F]FDG for PET imaging of arterial inflammation among PLHIV. To achieve an objective comparison, we applied the optimized imaging criteria for arterial inflammation recommended by the Cardiovascular Committee of the European Association of Nuclear Medicine (EANM) for [^18^F]FDG PET imaging [9].

## 2. Material and Methods

Between September 2019 and October 2020, we prospectively recruited HIV-infected patients who had been undergoing ART for 24 months or more, had achieved undetectable plasma HIV viremia, and were without a history of change in their ARV regimen in the preceding six months. We collected detailed history regarding ASCVD risks in the patients. We also obtained serum levels of lipids, hemoglobin, and C-reactive protein (CRP). We documented the most recent CD4+ T-cell count in all qualifying patients. Patients were subsequently scheduled to undergo [^68^Ga]Ga-pentixafor and ^18^F-FDG PET/CT imaging withing two weeks. All participants gave a written informed consent prior to enrolment into this study. The study was conducted in accordance with the Declaration of Helsinki, and the study protocol was approved by the Research Ethics Committee of the Faculty of Health Sciences, University of Pretoria (approval number:242/2018).

### 2.1. ^18^F-FDG PET: Image Acquisition and Analysis

Standard patient preparation was observed including a minimum of six hours of fasting. Blood glucose was less than 7.1 mmol/L at the time of [^18^F]FDG administration. Sixty minutes after intravenous administration of 3–5 MBq/Kg of [^18^F]FDG, vertex to mid-thigh CT followed PET imaging was acquired using standard oncologic PET imaging parameters on a Biograph 40 hybrid PET/CT scanner (Siemens Healthineers, Erlangen, Germany). This early PET/CT imaging was followed by a delayed imaging commenced at 120 min post [^18^F]FDG injection. The delayed imaging was optimized for vascular inflammation imaging as recommended by the Cardiovascular Committee of the EANM and as we have previously reported [9,10]. Briefly, PET/CT imaging was acquired from the base of skull to the level of the diaphragm at 8 min/bed position in 3D mode.

Image analysis was performed on a dedicated workstation equipped with Syngo.via software, VA60C (Siemens Healthineers, Erlangen, Germany) as previously reported [10]. Briefly, we obtained the maximum standardized uptake value (SUVmax) of arterial [^18^F]FDG uptake in the ascending aorta and the common carotid artery by averaging values obtained from multiple regions of interest (ROIs) in the arterial wall and lumen. For background activity correction, we averaged the SUVmean obtained from multiple ROIs from within the lumen of the superior vena cava (SVC) and internal jugular vein (IJV). We obtained a target-to-background ratio (TBR) for the ascending aorta and the carotid artery by dividing the aortic SUVmax by the SVC SUVmean and carotid SUVmax by IJV SUVmean, respectively. These parameters were obtained on the early (early-aortic-SUVmax, early-SVC-SUVmean, early-aortic-TBR, early-carotid-SUVmax, early-IJV-SUVmean, and early-carotid-TBR) and delayed (late-aortic-SUVmax, late-SVC-SUVmean, late-aortic-TBR, late-carotid-SUVmax, late-IJV-SUVmean, and late-carotid-TBR) ^18^F-FDG PET imagings.

### 2.2. [^68^Ga]Ga-Pentixafor: Tracer Synthesis, PET Imaging, and Image Analysis

An 1850 MBq loaded ^68^Ge/^68^Ga generator (iThemba LABS, Somerset West, South Africa) was used in-house to provide ^68^Ga-radioactivity to be labelled with precursor pentixafor (ABX advanced biochemical compounds, Biomedizinsche Forschugsreagenzien GmbH, Radeberg, Germany). A buffered solution of ^68^Ga radioactivity (1 mL) was labelled with pentixafor (50 µg). The reaction mixture at pH 3.5–4 was heated in a heating block (95 °C) for 10 min. Post incubation, radiolabeled [^68^Ga]Ga-pentixafor was purified using solid phase extraction (Sep Pack C-18-light cartridge (Waters Corporation, Massachusetts, USA)). An instant thin layer chromatography (ITLC) was performed to ascertain the radiochemical purity of the labeled product before administration into patients. A sample (5 µl) of [^68^Ga]Ga-pentixafor was spotted on the ITLC strip (ITLC-silica gel paper (Agilent, Forrest Lake, USA)) and developed in a mobile phase solution (Sodium Citrate (pH-5)). The developed ITLC strip (radioactivity) was counted and recorded on radio-chromatogram ((R*f* = 0.0–0.2 ([^68^Ga]Ga-pentixafor (>95%)), R*f* = 0.8–1.0 (free ^68^Ga/^68^Ga-colloids (<5%))).

Sixty minutes after intravenous administration of [^68^Ga]Ga-pentixafor, vertex to mid-thigh PET/CT imaging was acquired. No special patient preparation was observed before PET imaging. PET imaging was done in 3D mode at 3 min/bed position.

Image analysis was similar to that described for ^18^F-FDG PET imaging. Only a 60-min [^68^Ga]Ga-pentixafor PET/CT imaging was obtained. Aortic-SUVmax, SVC-SUVmean, aortic-TBR, carotid-SUVmax, IJV-SUVmean, and carotid-TBR were obtained on [^68^Ga]Ga-pentixafor PET/CT images.

### 2.3. Statistical Analysis

We performed descriptive statistics of baseline clinical and demographic information of patients. Categorical data are presented as frequencies while continuous variables are presented as mean ± standard deviation (SD) or as median (interquartile range, IQR). We compared the early and late [^18^F]FDG PET variables using Paired Sample *t*-test. We used Bland and Altman plots to show the level of agreement in arterial tracer quantification variables derived from [^18^F]FDG PET versus those derived from [^68^Ga]Ga-pentixafor PET imaging. We also used a Spearman correlation check for correlation between the arterial tracer quantification variables derived from [^18^F]FDG PET versus those derived from [^68^Ga]Ga-pentixafor PET imaging. Statistical significance was set at *p* < 0.05. We performed statistical analysis using IBM SPSS Statistics 21.0 (IBM Corp, Armonk, NY, USA).

## 3. Results

A total of 12 HIV-infected adults with suppressed HIV viremia were included, mean age was 44.67 ± 7.62 years. There were eight women (66.7%). The mean duration of HIV infection and mean CD+ T-cell count of the study population were 71.08 ± 37 months and 522.17 ± 260.33 cells/µL, respectively. The mean activity of administered [^18^F]FDG was significantly higher than that of [^68^Ga]Ga-pentixafor (7.84 ± 1.17mCi and 4.70 ± 2.12mCi, *p* < 0.001). The median interval between the two scans was 2 days (range = 1–11 days). Table 1 shows the details of the baseline clinical and demographic characteristics of the study population.

### 3.1. Comparison between Early and Late [^18^F]FDG PET Quantification Variables

Table 2 shows the details regarding comparison between the variables derived from the early and late scans. For the two arterial beds of interest, there was a significant increase in SUVmax in the late scan compared with the early scan. Venous blood pool activity showed a significant decrease between the early and the late scans as measured by venous SUVmean. The decline in SUVmean between the early and the late scans did not reach a statistical significance in the IJV. Consequent to an improvement in arterial SUVmax and a decline in venous SUVmean, there was a significant increase in the arterial TBR for both the aortic and carotid arterial beds.

### 3.2. Level of Agreement between [^18^F]FDG PET and [^68^Ga]Ga-Pentixafor PET-Derived Variables

**Figure 1:**
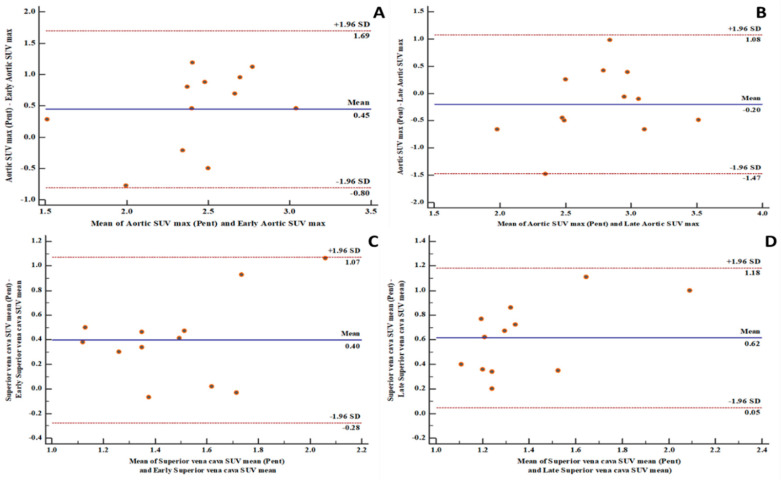
Bland and Altman plots showing good levels of agreement between fluorine-18 fluorodeoxyglucose positron emission tomography/computed tomography ([^18^F]FDG PET/CT) and Gallium-68pentixafor positron emission tomography/computed tomography ([^68^Ga]Ga-pentixafor PET/CT)-derived variables: (**A**) a good level of agreement between [^68^Ga]Ga-pentixafor PET-derived aortic maximum standardized uptake value (SUVmax) and [^18^F]FDG PET-derived early aortic SUVmax with 100% of measurements within the limits of agreement; (**B**) a good level of agreement between [^68^Ga]Ga-pentixafor PET-derived aortic SUVmax and [^18^F]FDG PET-derived late aortic SUVmax with 91.7% of measurements within the limits of agreement; (**C**) a good level of agreement between [^68^Ga]Ga-pentixafor PET-derived superior vena cava mean standardized uptake value (SUVmean) and [^18^F]FDG PET-derived early superior vena cava SUVmean with 100% of measurements within the limits of agreement; (**D**) a good level of agreement between [^68^Ga]Ga-pentixafor PET-derived superior vena cava SUVmean and [^18^F]FDG PET-derived late superior vena cava SUVmean with 100% of measurements within the limits of agreement. SD: standard deviation.

**Figure 2:**
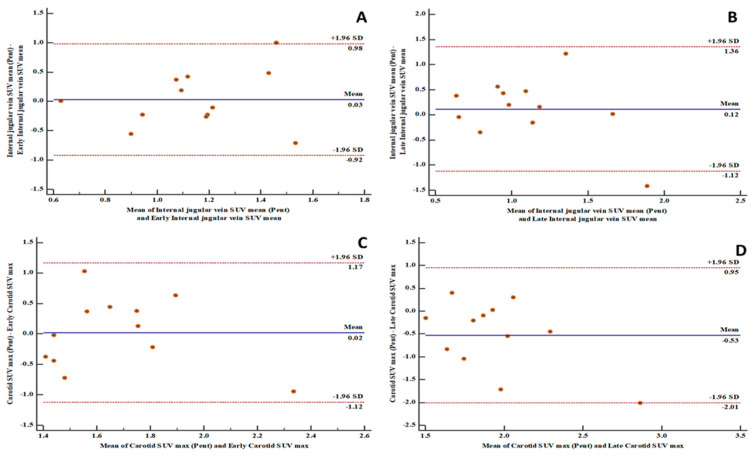
Bland and Altman plots showing good levels of agreement between [^18^F]FDG PET/CT and [^68^Ga]Ga-pentixafor PET/CT-derived variables: (**A**) a good level of agreement between [^68^Ga]Ga-pentixafor PET-derived internal jugular vein SUVmean and [^18^F]FDG PET-derived early internal jugular vein SUVmean with 91.7% of measurements within the limits of agreement; (**B**) a good level of agreement between [^68^Ga]Ga-pentixafor PET-derived internal jugular vein SUVmean and [^18^F]FDG PET-derived late internal jugular vein SUVmean with 91.7% of measurements within the limits of agreement; (**C**) a good level of agreement between [^68^Ga]Ga-pentixafor PET-derived carotid SUVmax and [^18^F]FDG PET-derived early carotid SUVmax with 100% of measurements within the limits of agreement; (**D**) a good level of agreement between [^68^Ga]Ga-pentixafor PET-derived carotid SUVmax and [^18^F]FDG PET-derived late carotid SUVmax with 91.7% of measurements within the limits of agreement. SD: standard deviation.

**Figure 3:**
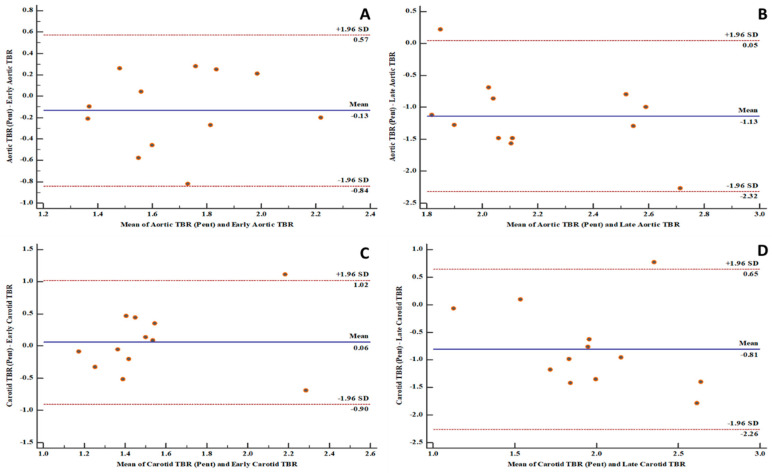
Bland and Altman plots showing good levels of agreement between fluorine-18 fluorodeoxyglucose positron emission tomography ([^18^F]FDG PET) and gallium-68 pentixafor positron emission tomography ([^68^Ga]Ga-pentixafor PET)-derived variables: (**A**) a good level of agreement between [^68^Ga]Ga-pentixafor PET-derived aortic target-to-background ratio (TBR) and [^18^F]FDG PET-derived early aortic target-to-background ratio (TBR) with 91.7% of measurements within the limits of agreement; (**B**) a good level of agreement between [^68^Ga]Ga-pentixafor PET-derived aortic target-to-background ratio (TBR) and [^18^F]FDG PET-derived late aortic target-to-background ratio (TBR) with 91.7% of measurements within the limits of agreement; (**C**) a good level of agreement between [^68^Ga]Ga-pentixafor PET-derived carotid target-to-background ratio (TBR) and [^18^F]FDG PET-derived early carotid target-to-background ratio (TBR) with 91.7% of measurements within the limits of agreement; (**D**) a good level of agreement between [^68^Ga]Ga-pentixafor PET-derived carotid target-to-background ratio (TBR) and [^18^F]FDG PET-derived late carotid target-to-background ratio (TBR) with 91.7% of measurements within the limits of agreement.

There is a high level of agreement between the [^18^F]FDG PET-derived quantification variables and the [^68^Ga]Ga-pentixafor PET-derived variables in the aortic and carotid arterial beds (Figure 1, Figure 2 and Figure 3). The level of agreement was higher for early [^18^F]FDG PET-derived quantification variables and [^68^Ga]Ga-pentixafor PET-derived variables than for the comparison between late [^18^F]FDG PET-derived quantification variables and [^68^Ga]Ga-pentixafor PET-derived variables.

### 3.3. Correlation between [^18^F]FDG PET and [^68^Ga]Ga-Pentixafor PET-Derived Variables

In the aortic arterial bed, we found a positive correlation between early and late aortic SUVmax, SVC SUVmean, aortic TBR derived from [^18^F]FDG PET versus aortic SUVmax, SVC SUVmean, and aortic TBR derived from [^68^Ga]Ga-pentixafor (Table 3). None of these positive correlations reached a statistical significance. In the carotid arterial bed, we found a negative correlation between early and late carotid SUVmax and early IJV SUVmean derived from [^18^F]FDG PET versus carotid SUVmax and IJV SUVmean derived from [^68^Ga]Ga-pentixafor PET/CT. In contrast, we found positive correlations between late IJV SUVmean and early and late carotid TBR derived from [^18^F]FDG PET/CT and IJV SUVmean and carotid TBR derived from [^68^Ga]-Ga-pentixafor PET/CT (Table 3). Similarly to the findings in the aortic arterial bed, these correlations did not reach statistical significance.

## 4. Discussion

[^18^F]FDG PET/CT is the most used radionuclide imaging technique for arterial inflammation imaging. In this prospective study, we evaluated the level of agreement in arterial tracer uptake quantification between [^18^F]FDG PET/CT and [^68^Ga]Ga-pentixafor PET/CT. We found a high level of agreement between the quantitative parameters obtained on both imaging techniques. We measured the level of agreements in the measurements of arterial tracer uptake obtained on both [^18^F]FDG PET/CT and [^68^Ga]Ga-pentixafor PET/CT using the Bland and Altman plots. We found 91 to 100% of measurements obtained on both scans were within a 1.96 standard deviation. The measurement of all variables obtained from both scans were equally distributed on either side of the zero line, indicating good consistency in the arterial tracer uptake quantification obtained from [^18^F]FDG PET/CT compared with that of [^68^Ga]Ga-pentixafor PET/CT. Our findings suggest that [^68^Ga]Ga-pentixafor may be as effective in quantifying arterial inflammation as is [^18^F]FDG PET/CT without experiencing the challenges associated with clinical [^18^F]FDG PET/CT imaging. We chose to make the comparison in PLHIV because of their elevated risk of ASCVD [1]. PLHIV represents a group where arterial inflammation is present from a young age even in the absence of traditional ASCVD risk factors and despite effective ART [6].

**Figure 4:**
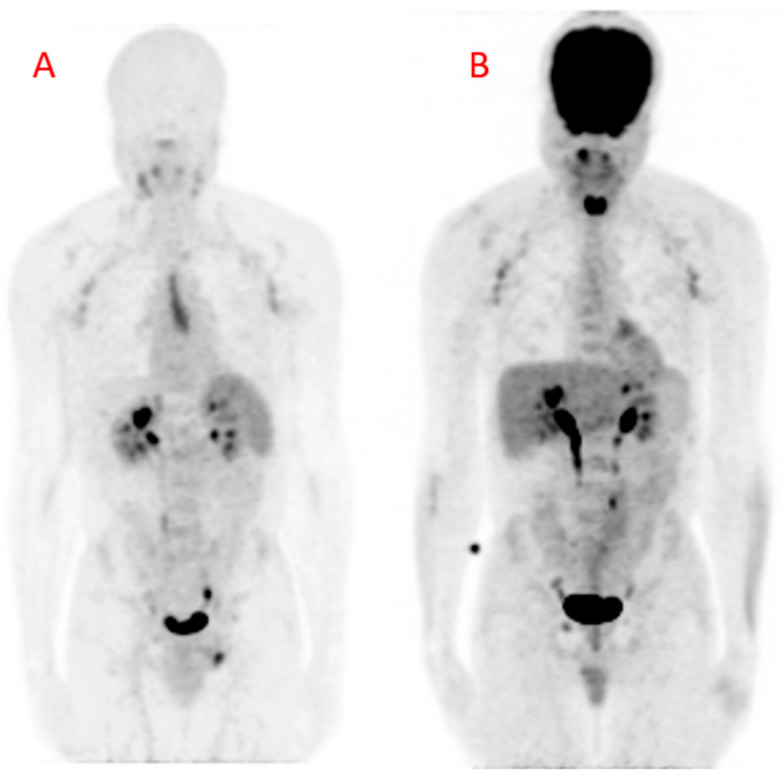
Maximum intensity projection (MIP) images obtained from (**A**) Gallium-68 pentixafor positron emission tomography ([^68^Ga]Ga-pentixafor PET) and (**B**) fluorine-18 fluorodeoxyglucose positron emission tomography (^18^[F]FDG PET) in a 42-year-old HIV-infected male. There are multiple mildly avid axillary lymphadenopathies and a urinary route of excretion of tracers on both images. The [^68^Ga]Ga-pentixafor PET image (**A**) shows physiologic tracer uptake in the spleen. The midline area of linear tracer uptake is localized to the thymus, likely related to inflammation associated with combination antiretroviral therapy (cART)-induced immune restoration. The [^18^F]FDG PET image (**B**) shows intense tracer uptake in the brain and the nasopharynx. There is also intense [^18^F]FDG in the muscles of phonation because the patient was talking during the period of uptake. This intense uptake can cause photon spill-over into the carotids during tracer uptake quantification. The patient arrived for the [^18^F]FDG PET/CT carrying a bag causing the diffuse [^18^F]FDG uptake in the lateral aspects of the forearms, more pronounced on the left. There is only minimal myocardial [^18^F]FDG uptake due to prolonged fasting in this patient.

Some important studies have shown the utility of [^18^F]FDG PET/CT in quantifying arterial inflammation in PLHIV [11,12]. [^18^F]FDG PET/CT studies have shown elevated arterial inflammation in PLHIV compared with age- and gender-matched controls [5,6]. In another study, [^18^F]FDG PET/CT was used to demonstrate residual arterial inflammation in patients who were treated with effective ART [13]. More recently, Hsue and colleagues demonstrated the utility of [^18^F]FDG PET/CT for treatment response assessment in HIV-infected people treated with canakinumab, an interleukin-1β inhibitor targeting arterial inflammation [14]. Some other studies have failed to show a significantly higher burden of arterial inflammation in PLHIV compared with non-HIV infected controls [15,16,17]. These contradictory findings may be related to the limitations associated with [^18^F]FDG PET/CT for arterial inflammation imaging [18]. Apart from the need for patients to fast and stop certain medications that can interfere with [^18^F]FDG biodistribution, there are important hurdles encountered when using [^18^F]FDG PET/CT for arterial inflammation quantification. The intense [^18^F]FDG accumulation in the myocardium and soft tissues of the neck precludes accurate arterial ^18^F-FDG uptake quantification in the coronary and carotid arteries, two of the most important arterial beds involved in ASCVD (Figure 4). During arterial [^18^F]FDG quantification for vascular inflammation imaging, the goal is to quantify [^18^F]FDG uptake by inflammatory cells. In ASCVD, [^18^F]FDG uptake in the vascular smooth muscle contributes significantly to the PET signal recorded during quantification [19].

In view of the limitations associated with the use of [^18^F]FDG PET/CT for arterial inflammation quantification, it is therefore important to evaluate other tracers for this indication. We chose [^68^Ga]Ga-pentixafor due to its specific binding to macrophages and not to other components of the arterial wall [7]. In addition, [^68^Ga]Ga-pentixafor does not show high physiologic uptake in multiple organs like ^18^F-FDG does. In the study by Hyafil and colleagues, only a few organs, including spleen, adrenals, and bone marrow, show significant physiologic [^68^Ga]Ga-pentixafor uptake. This allows for accurate quantification of tracer uptake in any arterial bed of interest. High expression of CXCR-4 in high-risk atheromatous lesions and precursor pre-atheromatous lesions makes [^68^Ga]Ga-pentixafor a viable tool for non-invasive imaging of arterial inflammation [20]. Good reproducibility of TBR quantification in atheromatous plaques makes [^68^Ga]Ga-pentixafor PET/CT a technically feasible modality for arterial inflammation imaging [21].

PET imaging of arterial inflammation presents a peculiar limitation due to the limited spatial resolution of the PET system causing significant underestimation of quantified arterial tracer uptake. To reduce the degree of this under-estimation, the Cardiovascular Committee of the EANM proposed certain imaging conditions to be observed while applying [^18^F]FDG PET/CT for atherosclerotic vascular inflammation imaging. In this study, we performed a dual-time-point imaging, using the routine oncologic imaging parameters, and subsequently applied the EANM recommendations to achieve a robust comparison of arterial quantification with both tracers evaluated in this study. While there was a significant increase in arterial [^18^F]FDG uptake and reduction in background blood-pool activity between the early and late imaging, the quantified [^68^Ga]Ga-pentixafor showed a good level of agreement with the [^18^F]FDG PET quantification parameters obtained on the early and late imaging. A study has previously evaluated the correlation between [^18^F]FDG PET/CT and [^68^Ga]Ga-pentixafor PET/CT in the quantification of tracer uptake in arterial lesions [22]. This study was a retrospective analysis of data obtained in oncologic patients imaged with both tracers. Similar to the results of our study, the authors also reported a weak correlation, albeit with a statistical significance. Our study differs from this previous study regarding the study population (we studied HIV-infected patients while the previous study included HIV-uninfected oncologic patients) and the optimization of the [^18^F]FDG PET imaging criteria applied in a prospective setting in our study. The weak and statistical insignificant correlations seen in our study despite a good agreement in quantification parameters between the two imaging modalities may be related to the differences in target between the two tracers. There is significant [^18^F]FDG uptake in all components of the arterial wall [19], while [^68^Ga]Ga-pentixafor is more specific for macrophages within the vascular endothelium [7]. Another possible contributor to a lack of statistical significance in the level of correlation in quantification parameters between the two imaging modalities may be related to the significant difference in the activity of tracer injected for the two imaging studies.

As is done for [^18^F]FDG PET/CT imaging of atherosclerotic vascular inflammation, it may be necessary to perform similar standardization for [^68^Ga]Ga-pentixafor PET/CT imaging of arterial inflammation imaging. This standardization may reduce the under-estimation of arterial tracer quantification. Respiratory and cardiac motion causes image blurring and results in under-estimation in PET signal quantification due to partial volume averaging. Gating of PET acquisition may be a viable way to standardize vascular inflammation imaging. This was confirmed in the study by Derlin et al. where the highest number of coronary lesions were seen in dual-gated imaging (corrected for respiratory and cardiac motion) compared with single or ungated [^68^Ga]Ga-pentixafor PET acquisition [23].

The strength of our study lies in its novelty. We performed a head-to-head comparison of [^18^F]FDG PET/CT and [^68^Ga]Ga-pentixafor PET/CT for arterial inflammation using standardized imaging criteria for the [^18^F]FDG PET/CT imaging. Our study included PLHIV, a group of patients in which ASCVD is a rising cause of morbidity and mortality but there is limited data showing the role of radionuclide imaging for disease characterization [24]. Our study has some limitations, the most important being the small study population. Despite this limited study population, we were able to demonstrate a high level of agreement between the two imaging modalities. Our study population has a low prevalence of ASCVD risks. PLHIV are at risk of ASCVD even in the absence of a high traditional cardiovascular risks.

## 5. Conclusions

There is a high level of agreement in the vascular tracer quantification as a marker of arterial inflammation between ^18^F-FDG PET/CT and [^68^Ga]Ga-pentixafor PET/CT in people living with HIV. We found no significant correlation in the quantification variables between the two imaging modalities. Optimization of imaging conditions for [^18^F]FDG PET/CT improves the level of arterial tracer uptake and background blood-pool clearance.

## Figures and Tables

**Table 1:** Demographic and clinical variables of the study population.

Variable	Frequency	Percent (%)
Age (years)		
Mean ± SD	44.67 ± 7.62	
Range	38–65	
Gender		
Male	4	33.3
Female	8	66.7
BMI		
Underweight	1	8.3
Normal	7	58.3
Overweight	3	25.0
Obese	1	8.3
Mean ± SD	24.18 ± 3.45	
Range	17.84–32.07	
Smoking		
Yes	1	8.3
No	11	91.7
HTN		
Yes	2	16.7
No	10	83.3
DM		
Yes	1	8.3
No	11	91.7
ART		
2NRTI + 1INSTI	1	8.3
2NRTI + 1NNRTI	9	75.0
2NRTI + 1PI	2	16.7
Family history of CVD		
Yes	2	16.7
No	10	83.3
FRS risk		
Low	8	66.7
Moderate	3	25.0
High	1	8.3
	Mean ± SD	
CD4+ T-cell count (cells/µL)	522.17 ± 260.33	
D:A:D risk score	2.59 ± 2.35	
C-reactive protein	11.08 ± 4.72	
Hemoglobin (g/dL)	11.59 ± 1.96	
FBS (mmol/L)	5.88 ± 1.51	
Lipid profile		
Triglyceride (mmol/L)	0.91 ± 0.21	
LDL cholesterol (mmol/L)	2.71 ± 0.56	
HDL cholesterol (mmol/L)	1.23 ± 0.19	
Total cholesterol (mmol/L)	4.33 ± 0.83	

BMI: Body Mass Index; HTN: Hypertension; DM: Diabetes Mellitus; ART: Antiretroviral Therapy; NRTI: Nucleoside Reverse Transcriptase Inhibitor; NNRTI: Non-Nucleoside Reverse Transcriptase Inhibitor; PI: Protease Inhibitor; INTSTI: Integrase Strand Transfer Inhibitor; CVD: Cardiovascular Disease; FRS: Framingham Risk Score; LDL: Low-Density Lipoprotein; HDL: High-Density Lipoprotein; FBS: Fasting Blood Sugar.

**Table 2:** Comparison between early and late [^18^F]FDG PET/CT-derived parameters.

Variable	Early	Late	*t*	*p*-Value
	Mean ± SD	Mean ± SD		
Aortic SUVmax	2.21 ± 0.39	2.85 ± 0.45	−5.160	<0.001 *
Superior vena cava SUVmean	1.28 ± 0.26	1.06 ± 0.22	2.701	0.021 *
Aortic TBR	1.76 ± 0.30	2.76 ± 0.52	−5.783	<0.001 *
Carotid SUVmax	1.66 ± 0.43	2.21 ± 0.64	−4.074	0.002 *
Internal jugular vein SUVmean	1.13 ± 0.31	1.05 ± 0.58	0.765	0.460
Carotid TBR	1.51 ± 0.38	2.38 ± 0.66	−4.741	0.001 *

*t*: Paired Samples *t*-test; *: *p*-value < 0.05 (i.e., statistically significant); SUVmax: maximum Standardized Uptake Value; SUVmean: mean Standardized Uptake Value; TBR: Target-to-Background Ratio.

**Table 3:** Correlation between [^18^F]FDG PET and [^68^Ga]Ga-pentixafor PET-derived variables.

[^18^F]FDG PET/CT	[^68^Ga]Ga-pentixafor PET/CT	
	*r*	*p*-Value
	Aortic SUVmax	
Early Aortic SUVmax	0.077	0.812
Late Aortic SUVmax	0.189	0.557
	Superior vena cava SUVmean	
Early Superior vena cava SUVmean	0.560	0.058
Late Superior vena cava SUVmean	0.235	0.463
	Aortic TBR	
Early Aortic TBR	0.344	0.274
Late Aortic TBR	0.225	0.483
	Carotid SUV max	
Early Carotid SUVmax	–0.403	0.194
Late Carotid SUVmax	–0.200	0.532
	Internal jugular vein SUVmean	
Early Internal jugular vein SUVmean	–0.168	0.602
Late Internal jugular vein SUVmean	0.217	0.499
	Carotid TBR	
Early Carotid TBR	0.123	0.704
Late Carotid TBR	0.295	0.352

*r*: Spearman Correlation Coefficient; SUVmax: maximum Standardized Uptake Value; SUVmean: mean Standardized Uptake Value; TBR: Target-to-Background Ratio.
